# Aortic root diameter is associated with HLA-B27: identifying the patient with ankylosing spondylitis at risk for aortic valve regurgitation

**DOI:** 10.1007/s00296-021-05040-w

**Published:** 2021-11-02

**Authors:** M. Baniaamam, S. C. Heslinga, T. C. Konings, M. L. Handoko, O. Kamp, V. P. van Halm, I. E. van der Horst-Bruinsma, M. T. Nurmohamed

**Affiliations:** 1 Amsterdam Rheumatology and Immunology Center, Reade Rheumatology, Jan van Breemenstraat 2, 1056 AB Amsterdam, The Netherlands; 2 Amsterdam Cardiovascular Sciences, Amsterdam, The Netherlands; 3 Department of Rheumatology, Amsterdam UMC, Vrije Universiteit Amsterdam, Amsterdam, The Netherlands; 4 Department of Cardiology, Amsterdam UMC, Vrije Universiteit Amsterdam, Amsterdam, The Netherlands; 5 Amsterdam Rheumatology and Immunology Center, Amsterdam UMC, Vrije Universiteit Amsterdam, Amsterdam, The Netherlands

**Keywords:** Ankylosing spondylitis, HLA-B27 genotype, Cardiovascular disease, Aortic root diameter, Transthoracic echocardiography

## Abstract

To assess the association between the aortic root diameter in HLA-B27 positive (+) and HLA-B27 negative (−) ankylosing spondylitis (AS) patients from the CARDAS cohort. The CARDAS study is a cross-sectional study in AS patients between 50 and 75 years who were recruited from a large rheumatology outpatient clinic. Patients underwent cardiovascular screening including echocardiography, with 2D, spectral, and color flow Doppler measurements. The aortic root was measured at sinuses of Valsalva during diastole. The aortic root diameter was adjusted for body surface area (BSA) (aortic root index, cm/m^2^). 193 Consecutive AS patients were included of whom 158 (82%) were HLA-B27 positive. The aortic root index was significantly higher in HLA-B27 + patients compared to HLA-B27− patients, respectively, 1.76 cm ± 0.21 vs. 1.64 cm ± 0.14, *p* < 0.001. No difference was seen in the prevalence of aortic valve regurgitation (AVR), *p* = 0.8. Regression analysis showed a significant association between HLA-B27 and aortic root index corrected for age, sex and cardiovascular risk factors (β 0.091, 95% CI 0.015–0.168, *p* = 0.02). Especially, male HLA-B27 + patients had a significantly increased aortic root index compared to male HLA-B27− AS patients, respectively, 1.76 cm (1.63–1.88) and 1.59 cm (1.53–1.68), *p* < 0.001. We found an increased aortic root index in elderly HLA-B27 + AS patients compared to HLA-B27− AS patients, especially in male patients. No difference was seen in the prevalence of AVR. However, as AVR can be progressive, echocardiographic monitoring in elderly male HLA-B27 + AS might be considered.

## Introduction

Ankylosing spondylitis (AS) is an inflammatory joint disease associated with cardiac involvement. Aortic valve regurgitation (AVR) is a well-known cardiac complication described in AS patients [1, 2]. Inflammation of the aortic root might weaken aortic wall strength with subsequent dilatation of the aortic root causing fibrotic thickening of the aortic cusps. In combination with shortening of the aortic cusps this might ultimately lead to AVR [3–5]. In the majority of patients with AVR, the disease course is chronic and slowly progressive and in severe cases can lead to congestive heart failure, arrhythmia and sudden death [6–8]. The American Heart Association/American College of Cardiology (AHA/ACC) and the European society of cardiology (ESC) state that AVR is pathological, regardless of its grade of severity [8, 9]. Timely intervention with life style changes, pharmaceutical and/or surgical treatment of the aortic valve reduces the risk for severe complications [10]. Hence, follow-up is indicated in all cases of AVR [8, 9]. In this light, some advocate regular echocardiographic screening for AS patients [11]. However, the cost–benefit of echocardiographic screening in AS is currently unknown and to what extent AS specific (subclinical) cardiac pathology leads to clinically overt cardiovascular morbidity and mortality remains to be elucidated. Therefore, it is needed to identify a specific at risk AS population that might benefit from routine echocardiographic monitoring.

HLA-B27 genotype is associated with AS. Eighty to 90% of AS patients in the Western World, carry the HLA-B27 allele compared to a 5% prevalence in the general Western population [12]. Interestingly, the HLA-B27 genotype has also been associated with cardiac diseases including AVR in non-rheumatic patients [4, 5]. The question rises whether the increased risk for cardiac diseases in AS patients is linked to the HLA-B27 genotype. Therefore, the presence of the HLA-B27 genotype may be a screening feature to identify AS patients at risk for AVR. Hence, we hypothesized that HLA-B27 positive (+) AS patients have a higher risk of developing AVR compared to HLA-B27 negative (−) AS patients, and, therefore, echocardiographic screening of HLA-B27 + AS patients could be of added value.

## Patients and methods

### Study population

Data of AS patients of the Cardiac disease in Ankylosing Spondylitis (CARDAS) cohort were used. The CARDAS cohort included AS patients aged between 50 and 75 years old who had a comprehensive cardiovascular screening. Subjects were consecutively recruited between March 2014 and September 2019 from the Rheumatology Department of Reade, Amsterdam, and the Netherlands. All patients fulfilled the 1984 Modified New York criteria for AS [13]. This study was conducted in accordance with the Helsinki Declaration and the approval was obtained from the ethics committee of the Slotervaart Hospital and Reade, Amsterdam, The Netherlands (NL44202.048.13). All participating patients gave written consent.

### Patient and disease characteristics

All patients underwent a complete AS workup and cardiovascular screening including transthoracic echocardiographic (TTE) screening. Medical history was obtained by questionnaires and medical charts, including medication use, cardiovascular risk factors including smoking, hypertension, and dyslipidemia and history of cardiovascular disease. Hypertension was defined as present if a patient was treated with antihypertensive medication, had a systolic blood pressure ≥ 140 mmHg or a diastolic blood pressure ≥ 90 mmHg measured during physical examination. Furthermore, physical examination included height, weight, and blood pressure measurements. Body mass index (BMI) was calculated. Blood sample measurements included standard hematological assessment, C-reactive protein (CRP), and erythrocyte sedimentation rate (ESR). Disease activity was measured with the Bath Ankylosing Spondylitis Disease Activity Index (BASDAI), Bath Ankylosing Spondylitis Functional Index (BASFI), and the Ankylosing Spondylitis Disease Activity Score—CRP (ASDAS-CRP).

### Transthoracic echocardiography

TTE was performed by experienced echo technicians at the Amsterdam UMC, location VUmc, a European Society of Cardiology certified echo-lab. To exclude inter-observer variability, all recordings of echocardiography data from both AS patients and controls were stored digitally and were afterwards analysed offline by a single cardiologist (T.C.K.). TTE was performed according to the protocol based on the guidelines provided by European Association of Echocardiography. Evaluation of cardiac function consisted of 2D, spectral and color flow Doppler recordings. 2D recordings were performed in parasternal long- and short-axis views, and apical four-, three-, and two-chamber views. The aortic root was measured at sinuses of Valsalva during diastole. The aortic root diameter was corrected for body surface area (BSA) according to the Dubois method (aortic root index) [14]. An aortic root index of ≥ 2.1 cm/m^2^ was considered as aortic root dilatation [15, 16].

### Statistical analysis

For data analysis SPSS Version 24.0 (IBM Corp., Armonk, New York) was used. Demographic and disease characteristics were summarized using descriptive statistics. Distribution of data was displayed by histograms. Values are expressed as mean ± standard deviation (SD), median (interquartile range, IQR) or numbers (percentages, %) where appropriate. Independent samples *t* tests were used for comparisons of normally distributed continuous variables and the Mann–Whitney *U* test for non-normally distributed variables. The Chi-squared test or Fisher’s exact test (*n* < 10) was performed on dichotomous variables. Echocardiographic data were analysed using the unpaired samples *t* test or the Mann–Whitney *U* test, where appropriate. A level of *p* < 0.05 was considered statistically significant.

## Results

### Patient characteristics

In total, 193 consecutive AS patients were included of whom 158 were HLA-B27 + and 35 HLA-B27−. AS patients had, respectively, a mean age of 60 (± 7) years and 61 (± 7) years and 44 (28%) and 11 (31%) were female, respectively (Table 1). Both groups had a comparable disease duration (time since diagnosis), 36 (± 11) and 30 (± 14) years, and a moderate disease activity assessed with the ASDAS-CRP, 2.1 (± 1.0) and (2.3 ± 1.0) (Table 1). Furthermore, obesity and diabetes type II were more common in HLA-B27− AS patients, respectively, 18% vs 40% and 9% vs 20%. History for cardiovascular disease was similar in both groups.

**Table 1:** Baseline characteristics

Patient characteristics	HLA-B27 +	HLA-B27 −
*n*	158	35
Age, years	60 ± 7	61 ± 7
Sex, female	44 (28)	11 (31)
BSA, m^2^	1.91 ± 0.19	2.00 ± 0.20
BMI, m^2^/kg	26.2 ± 4.1	28.7 ± 3.9
Systolic blood pressure, mmHg	133 ± 16	136 ± 15
Diastolic blood pressure, mmHg	83 ± 7	86 ± 8
Disease activity and severity		
Disease duration, years	36 ± 11	30 ± 14
ESR, mm/h	7 (4–14)	9 (5–17)
CRP, mg/L	2.7 (1.2–7.6)	3.3 (1.1–8.1)
ASDAS-CRP	2.1 ± 1.0	2.3 ± 1.0
BASDAI	3.3 ± 2.2	4.0 ± 2.4
BASFI	3.6 ± 2.4	4.0 ± 2.6
Cardiovascular risk factors		
Smoking		
Current	33 (21)	6 (17)
Ever	111 (70)	23 (66)
Pack years, years	26.2 ± 14.0	26.4 ± 16.8
Hypertension		
Criteria	93 (59)	25 (71)
Patient history	59 (37)	17 (49)
Obesity	28 (18)	14 (40)
Hypercholesterolemia	28 (18)	8 (23)
Diabetes type II	15 (9)	7 (20)
History of cardiovascular disease		
Angina pectoris	2 (1)	3 (9)
Myocardial infarction	10 (6)	2 (6)
Stroke	5 (3)	2 (6)
TIA	3 (2)	1 (3)
CVA	2 (1)	1 (3)
Peripheral ischemia	1 (1)	0 (0)
CABG	6 (4)	2 (6)
Family history (first degree)		
AP/MI	19 (12)	5 (14)
CVA/TIA	11 (7)	3 (9)
Medication		
Lipid modifying drug	29 (18)	9 (26)
Antihypertensives	68 (43)	17 (49)
Biologic DMARDs (anti-TNF)	58 (37)	12 (34)

Values are displayed as mean ± standard deviation (SD), median (IQR) or frequencies with corresponding percentages (%)

*BSA *body surface area, *BMI *body mass index, *ESR *erythrocyte sedimentation rate, *CRP *C-reactive protein, *ASDAS *ankylosing spondylitis disease activity score, *BASDAI *bath ankylosing spondylitis disease activity score, *BASFI *bath ankylosing spondylitis functional index, *TIA *transient ischemic attack, *CVA *cerebral vascular accident, *CABG *coronary artery bypass graft, *AP *angina pectoris, *MI *myocardial infarction

*Significance level of *p* ≤ 0.05

### Aortic root measurement and valvular disease

The median aortic root index was significantly higher in HLA-B27 + patients compared to HLA-B27− patients: 1.76 (± 0.21) cm/m^2^ vs 1.64 (± 0.14) cm/m^2^, *p* < 0.001. Furthermore, HLA-B27 + AS patients compared to HLA-B27− AS patients had more often aortic root dilatation (≥ 2.1 cm/m^2^), 11 (8%) and 1 (3%), *p* = 0.3, respectively (Table 2). The presence of aortic root dilatation in HLA-B27 + AS patients was seen significantly more often in patients with aortic valve regurgitation, *p* = 0.003. The single case of aortic root dilatation in the HLA-B27− cohort had a mild AVR. Of the eleven cases of aortic root dilatation in HLA-B27 + AS patients four had no AVR, one had AVR, five had mild AVR, one severe AVR and in one AS patient had an aortic valve prosthesis. None of the AS patients had aortic valve stenosis and one AS patient had mitral valve stenosis.

**Table 2.** Echocardiographic parameters

Echocardiography	HLA-B27 +	HLA-B27 −	*p* value
Aortic root, cm (mean ±SD)	3.35 ± 0.43	3.25 ± 0.28	0.12
Aortic root index, cm/m² (mean ±SD)	1.76 ± 0.21	1.64 ± 0.14	< 0.001*
Aortic root dilatation (≥2.1 cm/m²) (*n*, %)	11 (8)	1 (3)	0.5
Aortic valve regurgitation (*n*, %)	34 (22)	8 (24)	0.8
Trace (*n*, %)	13 (8)	3 (9)	
Mild (*n*, %)	18 (12)	5 (15)	
Moderate (*n*, %)	4 (3)	0	
Severe (*n*, %)	1 (1)	0	
Prothesis (*n*, %)	1 (1)	0	
Mitral valve regurgitation (*n*, %)	52 (34)	17 (49)	0.25
Mild (*n*, %)	49 (32)	16 (46)	
Moderate (*n*, %)	3 (2)	1 (3)	
Severe (*n*, %)	0 (0)	0 (0)	
Prothesis (*n*, %)	0 (0)	0 (0)	

Values are displayed as mean ± standard deviation (SD) or frequencies with corresponding percentages (%). No cases of aortic valve stenosis and only one case of mitral valve stenosis were assessed

*Significance level of *p* ≤ 0.05

### Association between HLA-B27 and aortic root index

The results of the linear regression analysis (crude, adjusted and fully adjusted) are presented in Table 3. The relation between HLA-B27 genotype and the aortic root index remained significant in the fully adjusted model corrected for age, sex and cardiovascular risk factors, B 0.091 (95%CI 0.091–0.168), *p* = 0.02.

**Table 3:** Association of HLA-B27 genotype with the aortic root index

	Crude model		Adjusted model^a^		Fully adjusted model^b^	
	Beta	*p* value	Beta	*p* value	Beta	*p* value
Aortic root index, cm/m^2^	0.121 (0.045–0.198)	0.002*	0.123 (0.048–0.199)	0.002*	0.091 (0.015–0.168)	0.02*

Values are displayed as odds ratio with corresponding 95% confidence interval

*Significance level of *p* ≤ 0.05

^a^Adjusted for age and gender

^b^Adjusted for age, gender, systolic blood pressure, diastolic blood pressure, hypercholesterolemia, BMI, Diabetes Mellitus type 2 and family history of cardiovascular diseases

### Aortic root index, HLA-B27 genotype and sex

The differences in aortic root index and sex and HLA-B27 genotype are shown in Table 4. Male HLA-B27 + patients had a significant increased aortic root index compared to male HLA-B27− AS patients, respectively, 1.76 (1.63–1.88) and 1.59 (1.53–1.68), *p* < 0.001. No difference was seen in female HLA-B27 + and HLA-B27− AS patients, respectively, 1.66 (1.53–1.92) and 1.64 (1.54–1.83), *p* = 0.91. Furthermore, male HLA-B27 + AS patients also had an increased aortic root index compared to female HLA-B27 + AS patients.

**Table 4:** Gender differences in aortic root index, aortic root dilatation, and aortic valve regurgitation

	Male		*p* value	Female		*p* value
	HLA-B27 +	HLA-B27 −		HLA-B27 +	HLA-B27 −	
*n* =	114	24		44	11	
Aortic root index, cm/m^2^	1.76 (1.63–1.88)	1.59 (1.53–1.68)	< 0.001*	1.66 (1.53–1.92)	1.64 (1.54–1.83)	0.91
Aortic root dilatation, *n* (%)	7 (7)	0	0.35	4 (11)	1 (10)	1.00
Aortic valve regurgitation, *n* (%)	23 (20)	5 (23)	0.80	11 (26)	3 (27)	0.94

Values are displayed as median (IQR) or frequencies with corresponding percentages (%). No cases of aortic valve stenosis and only one case of mitral valve stenosis

*Significance level of *p* ≤ 0.05

## Discussion

In the present study, the association between HLA-B27 genotype and aortic root diameter in AS patients was investigated. HLA-B27 + AS patients had a significantly increased aortic root index compared to HLA-B27− AS patients. This association was not explained by difference in age, sex nor cardiovascular risk factors. The prevalence of AVR was, however, similar in both groups. Furthermore, aortic dilatation was seen more often in HLA-B27 + AS patients, albeit this reached no statistical significance. Finally, our data suggest a sex and HLA-B27 genotype linked difference in aortic root index as male HLA-B27 + patients had an increased aortic root index compared to the HLA-B27− male patients and the overall female AS patients.

The HLA-B27 antigen is assumed to misfold causing an unfolded protein response (UPR). This might result in IL-23 upregulation by macrophages and lamina propria mononuclear cells and leading to activation of the IL-23/IL-17 inflammatory pathway, increasing susceptibility to AS [17–19]. Pathology studies have shown that IL-23-Dependent γ/δ T-cells (IL23 + T-cell) are specifically expressed in entheseal organs such as entheses (the primary inflammatory target in AS), ciliary body of the eye, and aortic root and aortic valves [17, 20]. Entheseal organs are avascular in their fibrocartilaginous regions, however, microdamages of entheseal tissue due to continuous exposure to mechanical forces are common and appear to be associated with tissue repair responses and vessel ingrowth [21, 22]. It is assumed that enthesis-resident IL23 + T-cells have a homeostatic role in entheseal tissue repair. However, enthesis-resident IL23 + T-cells can also trigger a pro-inflammatory cascade as IL-23 binding induces IL-17 and IL-22 production leading to inflammation, bone loss, and ossification [17, 23]. Therefore, IL-23 upregulation possibly also results in activation of in situ IL23 + T-cells in aortic root and valves leading to local inflammation with subsequently inflammation of the aortic root and valves. In fact, murine studies have shown inflammation of entheses and aortic root and valves after infusion with IL-23 [20].

Our study partially confirms our hypothesis as HLA-B27 + AS patients did not have an increased prevalence of aortic valve disease as was expected. However, HLA-B27 + AS patients did have an increased aortic root index and had more often aortic root dilatation. To our knowledge, this finding has not been reported before. Our results may be indicative for the preclinical state of aortic valve disease in HLA-B27 + AS patients. At an older age HLA-B27 + AS patients may have a higher risk at developing aortic valve disease as inflammation of the aortic root and valves is progressive of nature [7]. Klingberg et al. found also no relation between HLA-B27 genotype and aortic valve regurgitation [11]. However, in this study assessment of this relation was not the primary objective and the AS subjects were 10 years younger. Moreover, Huppert et al. found, in HLA-B27 + juvenile arthritis patients, a higher prevalence of aortic valve regurgitation compared to HLA-B27− non-rheumatic controls, respectively, 10% (4/40) vs 0% (0/40) [24]. However, this study was performed in children < 18 years of age and the control group consisted of non-rheumatic HLA-B27− subjects.

Our data suggest a sex and HLA-B27 genotype linked difference in aortic root index in disadvantage for male HLA-B27 + patients. In this light, the findings of Bergfeldt et al. are important as they showed that patients needing a pacemaker due to severe conduction disturbances were primarily male HLA-B27 + patients [25].

Our study did have some limitations. First, due to the cross-sectional study design, the associations found in this study are not necessarily causal. Therefore, the (potential) long-term consequences of the cardiac manifestations we observed in our patients still need to be demonstrated. AS is predominant in male AS patients, therefore, the female subgroup was relatively small what could have masked differences between HLA-B27 + and HLA-B27 women.

In conclusion, our study demonstrated an increased aortic root index in elderly HLA-B27 + AS patients, especially in male patients. This did not already translated in an increased prevalence of aortic valve regurgitation in this subgroup. However, this finding is important as inflammation of the aortic root and valve in chronic inflammatory disease are progressive and may evolve in severe complications. Therefore, to recognize the AS patients at risk for this cardiac disease, echocardiographic monitoring, particularly in elderly male HLA-B27 + AS subjects should be considered. Obviously, prospective studies should determine the long-term outcomes as well the cost effectiveness of echocardiographic screening in this subgroup.

## Declarations

### Data sharing statement

The datasets generated during and/or analysed during the current study are available from the corresponding author on reasonable request.
